# Analysis of data collected in the European Society for Blood and Marrow Transplantation (EBMT) Registry on a cohort of lymphoma patients receiving plerixafor

**DOI:** 10.1038/s41409-019-0693-z

**Published:** 2019-09-30

**Authors:** Anna Sureda, Christian Chabannon, Tamás Masszi, David Pohlreich, Christof Scheid, Catherine Thieblemont, Björn E. Wahlin, Ioanna Sakellari, Nigel Russell, Andrea Janikova, Anna Dabrowska-Iwanicka, Cyrille Touzeau, Albert Esquirol, Esa Jantunen, Steffie van der Werf, Paul Bosman, Ariane Boumendil, Qianying Liu, Marina Celanovic, Silvia Montoto, Peter Dreger

**Affiliations:** 1 Institut Català d’Oncologia, Hospital Duran i Reynals, Barcelona, Spain; 2 Institut Paoli-Calmettes, Marseille, France; 3 Semmelweis University, Budapest, Hungary; 4 Charles University Hospital, Prague, Czech Republic; 5 University of Cologne, Cologne, Germany; 6 APHP, Hôpital Saint-Louis, Service d’hémato-oncologie, Université Paris Diderot - and Université Sorbonne Paris Cité, Paris, France; 7 Karolinska University Hospital, Stockholm, Sweden; 8 George Papanicolaou General Hospital, Thessaloniki, Greece; 9 Nottingham University Hospital, Nottingham, UK; 10 University Hospital Brno, Brno, Czech Republic; 11 Maria Sklodowska-Curie Institute-Oncology Center, Warsaw, Poland; 12 CHU Nantes, Nantes, France; 13 Hospital de la Santa Creu Sant Pau, Barcelona, Spain; 14 University of Eastern Finland and Kuopio University Hospital, Kuopio, Finland; 15 EBMT Data Office, Leiden, Netherlands; 16 EBMT Statistical Unit, Paris, France; 17 Sanofi Genzyme, Cambridge, MA USA; 18 St Bartholomew’s Hospital, Barts Health NHS Trust, London, UK; 19 University of Heidelberg, Heidelberg, Germany

**Keywords:** Stem-cell therapies, Immunopathogenesis

## Abstract

Plerixafor + granulocyte-colony stimulating factor (G-CSF) is administered to patients with lymphoma who are poor mobilizers of hematopoietic stem cells (HSCs) in Europe. This international, multicenter, non-interventional registry study (NCT01362972) evaluated long-term follow-up of patients with lymphoma who received plerixafor for HSC mobilization versus other mobilization methods. Propensity score matching was conducted to balance baseline characteristics between comparison groups. The following mobilization regimens were compared: G-CSF + plerixafor (G + P) versus G-CSF alone; G + P versus G-CSF + chemotherapy (G + C); and G-CSF + plerixafor + chemotherapy (G + P + C) versus G + C. The primary outcomes were progression-free survival (PFS), overall survival (OS), and cumulative incidence of relapse (CIR). Overall, 313/3749 (8.3%) eligible patients were mobilized with plerixafor-containing regimens. After propensity score matching, 70 versus 36 patients were matched in the G + P versus G-CSF alone cohort, 124 versus 124 in the G + P versus G + C cohort, and 130 versus 130 in the G + P + C versus G + C cohort. For both PFS and OS, the upper bound of confidence interval for the hazard ratio was >1.3 for all comparisons, implying that non-inferiority was not demonstrated. No major differences in PFS, OS, and CIR were observed between the plerixafor and comparison groups.

## Introduction

Lymphomas are the second most common indication for autologous hematopoietic stem cell (HSC) transplantation (HSCT). The most common method for mobilizing HSCs from the peripheral blood is treatment with granulocyte-colony stimulating factor (G-CSF) alone or combined with chemotherapy [1, 2]. However, a sizable minority of patients fail to mobilize sufficiently with G-CSF-based regimens [3, 4].

Plerixafor has a mode of action different from other HSC mobilizing agents and acts by binding to chemokine (C-X-C motif) receptor 4 (CXCR4), preventing the binding of its ligand stromal cell-derived factor 1 (SDF-1, now C-X-C motif chemokine 12, CXCL12) and thereby inhibiting events downstream of CXCL12 including SDF-1-mediated G-protein activation, receptor internalization, calcium flux, and chemotaxis [5, 6]. The CXCL12/CXCR4 interaction is an integral part of the mechanism of homing and retention of HSC in the bone marrow and inhibition of this interaction by plerixafor mobilizes HSCs from the bone marrow [7, 8]. Unlike cytokines used for HSC mobilization (e.g., G-CSF), plerixafor is not a growth factor and does not cause cell proliferation or expansion. Therefore, the approved use for plerixafor is in combination with G-CSF [9, 10].

As there is a theoretical risk of tumor cell mobilization with any stem cell mobilization method for HSCT, the European Medicines Agency requested a postapproval analysis of plerixafor to evaluate the possible long-term negative impact related to tumor cell mobilization.

The European Society for Blood and Marrow Transplantation (EBMT) database is run and accessed through a project manager internet server and is accessible to all EBMT registered centers. Our analysis of the EBMT registry data for long-term clinical outcomes included progression-free survival (PFS), overall survival (OS), and cumulative incidence of relapse (CIR) in patients with lymphoma. Patients who received plerixafor for mobilization of HSCs were matched by propensity scoring with patients who received other standard mobilization methods. Only patients who had a subsequent HSCT were included in the study.

## Methods

### Study design

This was an international, multicenter, non-interventional registry study, with patient follow-up ranging from 3.5 to 7.5 years, which evaluated the outcomes for patients with lymphoma who received G-CSF + plerixafor compared with other mobilization methods for HSC mobilization and who received an autologous HSCT (ClinicalTrials.gov number NCT01362972). All patients ≥18 years from the EBMT registry, with a diagnosis of lymphoma who were considered poor mobilizers and had undergone their first autologous HSCT between 2008 and 2012, were considered eligible for the study. Patients in the plerixafor groups who were poor mobilizers were those treated according to the label. Patients enrolled in the registry and evaluated in this study were from Austria, Belgium, Bulgaria, Czech Republic, Finland, France, Germany, Greece, Hungary, Ireland, Israel, Italy, Netherlands, Poland, Romania, Spain, Sweden, Switzerland, and the United Kingdom.

The study was conducted in accordance with the Declaration of Helsinki and the International Conference on Harmonization Guidelines for Good Clinical Practice. Approval of the protocol was obtained from all participating sites, local governmental authorities, and Institutional Review Boards. This was a non-inferiority study, with a non-inferiority margin of a 30% increase in PFS and OS corresponding to a hazard ratio (HR) upper bound of 1.3; no lower limit was set.

### Poor mobilizers

Predicted poor mobilizers were defined as patients who had received prior irradiation to marrow bearing areas or had high exposure to marrow-damaging chemotherapy. Proven poor mobilizers were defined as patients who in a previous mobilization attempt failed to mobilize sufficient CD34+ cells in peripheral blood to proceed to apheresis or to proceed to transplantation, or who, in the current mobilization, failed to achieve a sufficient increase in peripheral blood CD34+ cells at the predicted time for peak mobilization [11]. In this study, only poor mobilizers (either predicted or proven) have been considered in the propensity score matching and analyses.

### Data collection

The data were entered, managed, and maintained centrally in an internet accessible database. Variables present on the EBMT Minimum Essential Data A and B forms were used to derive the data for the study and Medical C form was used to obtain plerixafor data.

### Outcomes

The primary efficacy outcomes were OS, PFS, and CIR. Secondary efficacy outcomes were hematological recovery (time to achieve absolute neutrophil counts of ≥0.5 × 10^9^/L and platelet counts of ≥50 × 10^9^/L). All transplant complications occurring within 100 days of transplantation were recorded.

The following mobilization regimens were assessed:

- G-CSF + plerixafor versus G-CSF alone;
- G-CSF + plerixafor versus G-CSF + chemotherapy;
- G-CSF + plerixafor + chemotherapy versus G-CSF + chemotherapy.

Graft failure was defined as: (1) the loss of the graft with neutrophils reaching ≥ 0.5 × 10^9^ cells/L but subsequently decreasing to a lower level of cells until additional treatment for engraftment was provided, or (2) no engraftment, where neutrophils never reached ≥0.5 × 10^9^ cells/L.

### Statistical analyses

Due to the observational nature of the study, no formal statistical hypothesis testing was planned with adequate power or Type 1 error control.

Propensity score method was used to identify study comparison groups that were balanced with respect to baseline characteristics, including, demographics, lymphoma type, disease characteristics and staging, prior treatment characteristics, and disease status [12]. The baseline variables and patient demographics used for propensity score matching are shown in Table 1. Only patients who were identified as a “proven or predicted poor mobilizer” were included in the analysis.

**Table 1:** Patient demographics in the matched comparison groups used for propensity score matching

	Comparison 1		Comparison 2		Comparison 3	
	G-CSF + plerixafor (*N* = 70)	G-CSF alone (*N* = 36)	G-CSF + plerixafor (*N* = 124)	G-CSF + chemotherapy (*N* = 124)	G-CSF + plerixafor + chemotherapy (*N* = 130)	G-CSF + chemotherapy (*N* = 130)
Age at first mobilization, mean ± SD, years	(*N* = 65)	(*N* = 32)	(*N* = 117)	(*N* = 107)	(*N* = 130)	(*N* = 115)
	51.4 ± 13.5	51.2 ± 15.8	52.5 ± 12.6	51.5 ± 12.8	52.6 ± 13.6	52.3 ± 12.3
Females, *n* (%)	33 (47.1)	15 (41.7)	56 (45.2)	47 (37.9)	51 (39.2)	54 (41.5)
Bone marrow involvement at the start of mobilization, *n*/*N* (%)	2/66 (3.0)	2/33 (6.1)	8/104 (7.7)	4/102 (3.9)	8/112 (7.1)	4/105 (3.8)
Status of disease at collection, *n* (%)	*N* = 67	*N* = 34	*N* = 113	*N* = 108	*N* = 117	*N* = 115
Complete response 1 or partial response 1 or very good partial response	42 (62.7)	23 (67.6)	61 (54.0)	57 (52.8)	63 (53.8)	58 (50.4)
Refractory disease	2 (3.0)	1 (2.9)	14 (12.4)	9 (8.3)	15 (12.8)	12 (10.4)
Sensitive disease, complete response >1, partial response >1	23 (34.3)	10 (29.4)	38 (33.6)	42 (38.9)	39 (33.3)	45 (39.1)
Ann Arbor stage at diagnosis, *n* (%)	*N* = 65	*N* = 35	N = 111	*N* = 111	*N* = 112	*N* = 117
I	5 (7.7)	5 (14.3)	8 (7.2)	9 (8.1)	5 (4.5)	6 (5.1)
II	10 (15.4)	4 (11.4)	23 (20.7)	24 (21.6)	24 (21.4)	23 (19.7)
III	13 (20.0)	5 (14.3)	22 (19.8)	21 (18.9)	23 (20.5)	26 (22.2)
IV	37 (56.9)	21 (60.0)	58 (52.3)	57 (51.4)	60 (53.6)	62 (53.0)
Interval between diagnosis and first collection, mean ± SD, months	*N* = 65	*N* = 32	*N* = 117	*N* = 107	*N* = 130	*N* = 115
Year of first transplant	18.9 ± 20.2	16.3 ± 20.6	32.5 ± 48.1	30.6 ± 45.3	23.6 ± 28.7	23.7 ± 28.4
2009	20 (28.6)	12 (33.3)	32 (25.8)	39 (31.5)	21 (16.2)	34 (26.2)
2010	23 (32.9)	13 (36.1)	31 (25.0)	35 (28.2)	44 (33.8)	48 (36.9)
2011	16 (22.9)	7 (19.4)	37 (29.8)	39 (31.5)	46 (35.4)	37 (28.5)
2012	11 (15.7)	4 (11.1)	24 (19.4)	11 (8.9)	19 (14.6)	11 (8.5)
Geographic region, *n* (%)						
South and East Europe	28 (40.0)	18 (50.0)	52 (41.9)	42 (33.9)	43 (33.1)	41 (31.5)
North and West Europe	42 (60.0)	18 (50.0)	72 (58.1)	82 (66.1)	87 (66.9)	89 (68.5)
Lymphoma disease types, *n* (%)						
Hodgkin’s lymphoma	13 (18.4)	8 (22.2)	22 (17.7)	26 (21.0)	21 (16.2)	24 (18.5)
Aggressive B-cell lymphoma	24 (34.3)	12 (33.3)	43 (34.7)	44 (35.5)	56 (43.1)	52 (40.0)
Indolent B-cell lymphoma	12 (17.1)	6 (16.7)	21 (16.9)	23 (18.5)	25 (19.2)	24 (18.5)
Mantle cell lymphoma	20 (28.6)	9 (25.0)	26 (21.0)	19 (15.3)	13 (10.0)	14 (10.8)
T-cell lymphoma	1 (1.4)	1 (2.8)	12 (9.7)	12 (9.7)	14 (10.8)	14 (10.8)
Others^a^	0	0	0	0	1 (0.8)	2 (1.5)

*G-CSF* granulocyte-colony stimulating factor, *SD* standard deviation

^a^Not disclosed

A single imputation approach was implemented to create complete data sets for analyses. Propensity scores were then fit using logistic regression models. Matches for plerixafor patients were identified from the non-plerixafor groups based on the estimated propensity scores. Matching was performed without replacement. Model success was based on whether balance between the plerixafor and the control groups matched samples was achieved.

Following the propensity score analysis, the outcomes for each mobilization treatment group were analyzed for comparable groups. Cox proportional hazards model with covariates was used for OS and PFS. The 95% confidence intervals (CI) and HR for the effect of treatment were calculated. Potential covariates included: interval from diagnosis to transplantation, disease status at conditioning and conditioning regimen, and disease status at time of transplantation.

Survival curves were developed for each treatment group using nonparametric Kaplan–Meier estimates [13], as well as survival rates at 6 months, and at 1, 2, 3, 4, and 5 years.

A competing risk model was developed for CIR; death without prior progression/relapse was treated as a competing event. The 95% CI and cumulative incidence at each year post transplantation were estimated.

Sample size was estimated using the following assumptions: 15% of transplanted lymphoma patients would receive G-CSF alone and 85% would receive G-CSF + chemotherapy; 10% of patients transplanted from each regimen would receive plerixafor treatment; and 70% of patients receiving plerixafor would be matched at a ratio of 1:2 plerixafor to comparator.

## Results

### Participants and demographics

**Fig. 1:**
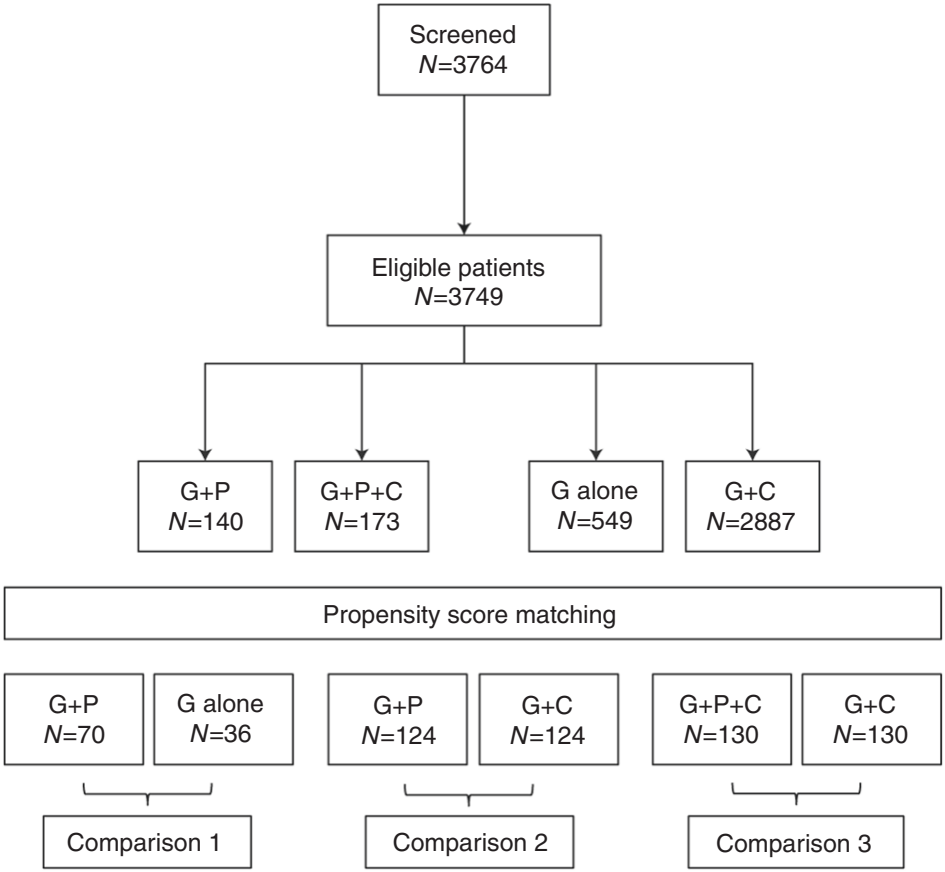
Patient eligibility and treatment. Key: G G-CSF, P plerixafor, C chemotherapy

Overall, 3764 patients were screened and 3749 were eligible to be included in the study (Fig. 1). These included 140 patients treated with G-CSF + plerixafor, 173 patients treated with G-CSF + chemotherapy + plerixafor, 549 patients treated with G-CSF alone, and 2887 patients treated with G-CSF + chemotherapy. The propensity score matching of predicted and proven poor mobilizers identified matched groups for the comparative analysis (Table 1). The number of patients classified as predicted or proven poor mobilizers was 136 treated with G-CSF + plerixafor, 173 treated with G-CSF + chemotherapy + plerixafor, 54 treated with G-CSF alone, and 245 treated with G-CSF + chemotherapy.

After propensity scoring, 70 patients in the G-CSF + plerixafor were matched with 36 patients in the G-CSF alone cohort, 124 matched with 124 in the G-CSF + plerixafor versus G-CSF + chemotherapy cohort, and 130 matched with 130 in the G-CSF + plerixafor + chemotherapy versus G-CSF + chemotherapy cohort. Disease history and baseline demographics of the patients are shown in Table 1.

The proportion of patients who were proven poor mobilizers was greater in the plerixafor cohorts (ranging from 97.1 to 98.4%) compared with the comparator cohorts (ranging from 68.3 to 75.0%). More patients in the plerixafor groups failed to mobilize sufficient CD34+ cells at the predicted peak mobilization time compared with patients in the comparator groups (Table 2).

**Table 2:** Mobilization characteristics for the matched comparator groups

	Comparison 1		Comparison 2		Comparison 3	
	G-CSF + plerixafor (*N* = 70)	G-CSF alone (*N* = 36)	G-CSF + plerixafor (*N* = 124)	G-CSF + chemotherapy (*N* = 124)	G-CSF + plerixafor + chemotherapy (*N* = 130)	G-CSF + chemotherapy (*N* = 130)
Predicted poor mobilizers,* *n*/*N* (%)	16/64 (25.0)	14/34 (41.2)	29/116 (25.0)	62/122 (50.8)	26/130 (20.0)	60/127 (47.2)
Proven poor mobilizers,** *n*/*N* (%)	68/70 (97.1)	27/36 (75.0)	120/122 (98.4)	84/123 (68.3)	127/130 (97.7)	89/129 (69.0)
Current mobilization						
Failed to mobilize sufficient CD34+ cells at predicted time, *n*/*N*	33/46 (71.7)	6/16 (37.5)	56/81 (69.1)	12/47 (25.5)	72/97 (74.2)	13/53 (24.5)
(%) CD34+ cell count, median × 10^9^/L	5.0 (*n* = 39)	16.0 (*n* = 10)	5.2 (*n* = 66)	8.9 (*n* = 16)	5.0 (*n* = 76)	8.8 (*n* = 17)

*G-CSF* granulocyte-colony stimulating factor, *SD* standard deviation

*Predicted poor mobilizers were defined as patients who had received prior irradiation to marrow bearing areas or had high exposure to marrow-damaging chemotherapy

**Proven poor mobilizers were defined as patients who in a previous mobilization attempt failed to mobilize sufficient CD34+ cells in the peripheral blood to proceed to apheresis or to proceed to transplantation or who, in the current mobilization, failed to achieve a sufficient increase in peripheral blood CD34+ cells at the predicted time for peak mobilization

### Primary outcomes

#### Progression-free survival

**Fig. 2:**
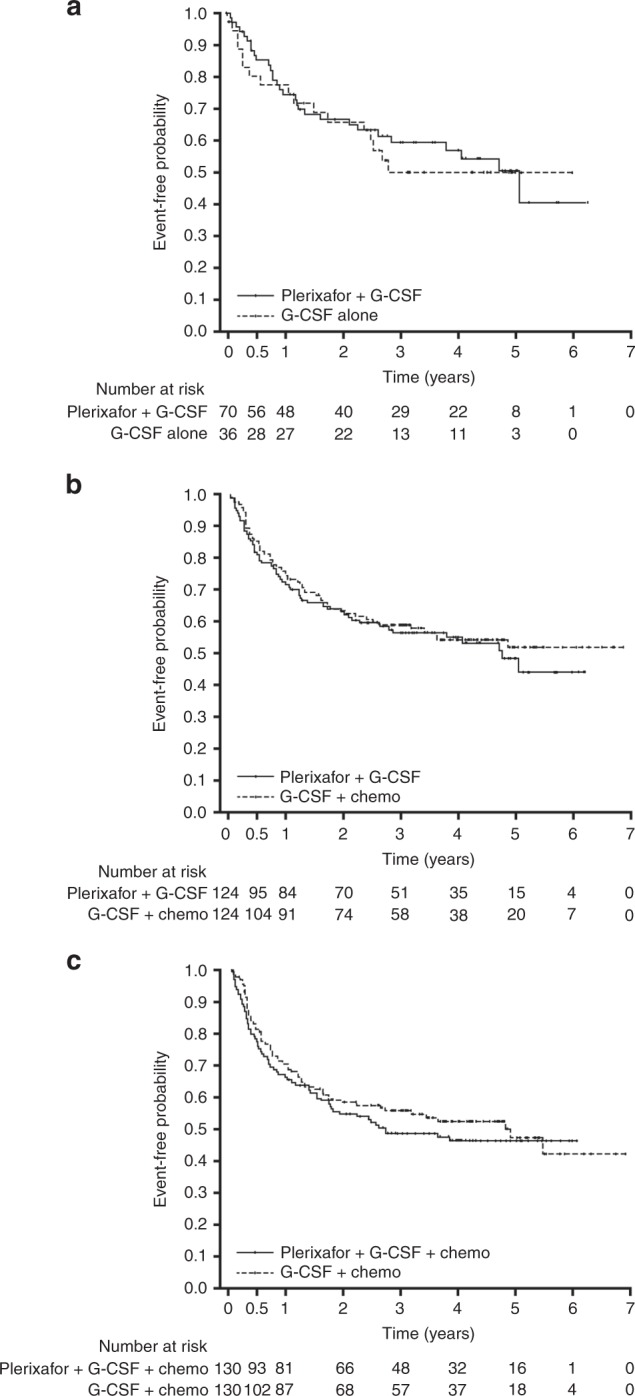
Progression-free survival for each of the comparison groups, **a** G-CSF + plerixafor versus G-CSF alone (comparison 1); **b** G-CSF + plerixafor versus G-CSF + chemotherapy (comparison 2); and **c** G-CSF + plerixafor + chemotherapy versus G-CSF + chemotherapy (comparison 3)

The estimated PFS at 3 years are shown in Table 3 and were similar between comparison groups. The Kaplan–Meier estimates for PFS were also similar for all the comparisons, G-CSF + plerixafor versus G-CSF alone (Fig. 2a), G-CSF + plerixafor versus G-CSF + chemotherapy (Fig. 2b), and G-CSF + plerixafor + chemotherapy versus G-CSF + chemotherapy (Fig. 2c).

**Table 3:** Primary outcomes progression-free survival, overall survival, and cumulative incidence of relapse for each of the comparator groups

	Comparison 1		Comparison 2		Comparison 3	
	G-CSF + plerixafor (*N* = 70)	G-CSF alone (*N* = 36)	G-CSF + plerixafor (*N* = 124)	G-CSF + chemotherapy (*N* = 124)	G-CSF + plerixafor + chemotherapy (*N* = 130)	G-CSF + chemotherapy (*N* = 130)
Estimated PFS, at 3 years, [95% CI]	0.59 [0.46, 0.70]	0.50 [0.32, 0.65]	0.56 [0.47, 0.65]	0.59 [0.50, 0.67]	0.48 [0.39, 0.57]	0.56 [0.46, 0.64]
PFS hazard ratio [95% CI]^a^	0.82 [0.44, 1.53]		1.16 [0.78, 1.73]		1.16 [0.80, 1.67]	
Estimated OS at 3 years, [95% CI]	0.75 [0.62, 0.84]	0.70 [0.51, 0.83]	0.69 [0.59, 0.76]	0.76 [0.68, 0.83]	0.67 [0.58, 0.75]	0.71 [0.62, 0.78]
OS hazard ratio [95% CI]^a^	0.97 [0.47, 2.03]		1.45 [0.91, 2.32]		1.29 [0.78, 2.11]	
Deaths, *n* (%)	20 (28.6)	11 (30.6)	41 (33.1)	31 (25.0)	45 (34.6)	38 (29.2)
Deaths Hazard ratio [95% CI]^a^	0.97 [0.47, 2.03]		1.45 [0.91, 2.32]		1.29 [0.78, 2.11]	
CIR, 3 years [95% CI]	0.33 [0.22, 0.45]	0.44 [0.27, 0.61]	0.38 [0.29, 0.47]	0.36 [0.28, 0.45]	0.44 [0.35, 0.52]	0.38 [0.30, 0.47]

*CI* confidence interval, *CIR* cumulative incidence of relapse, *G-CSF* granulocyte-colony stimulating factor, *OS* overall survival, *PFS* progression-free survival

^a^Hazard ratio with covariate adjustment including status of disease at time of transplant, conditioning regimens, and interval from diagnosis to transplantation and performance status (Karnofsky)

#### Overall survival

**Fig. 3:**
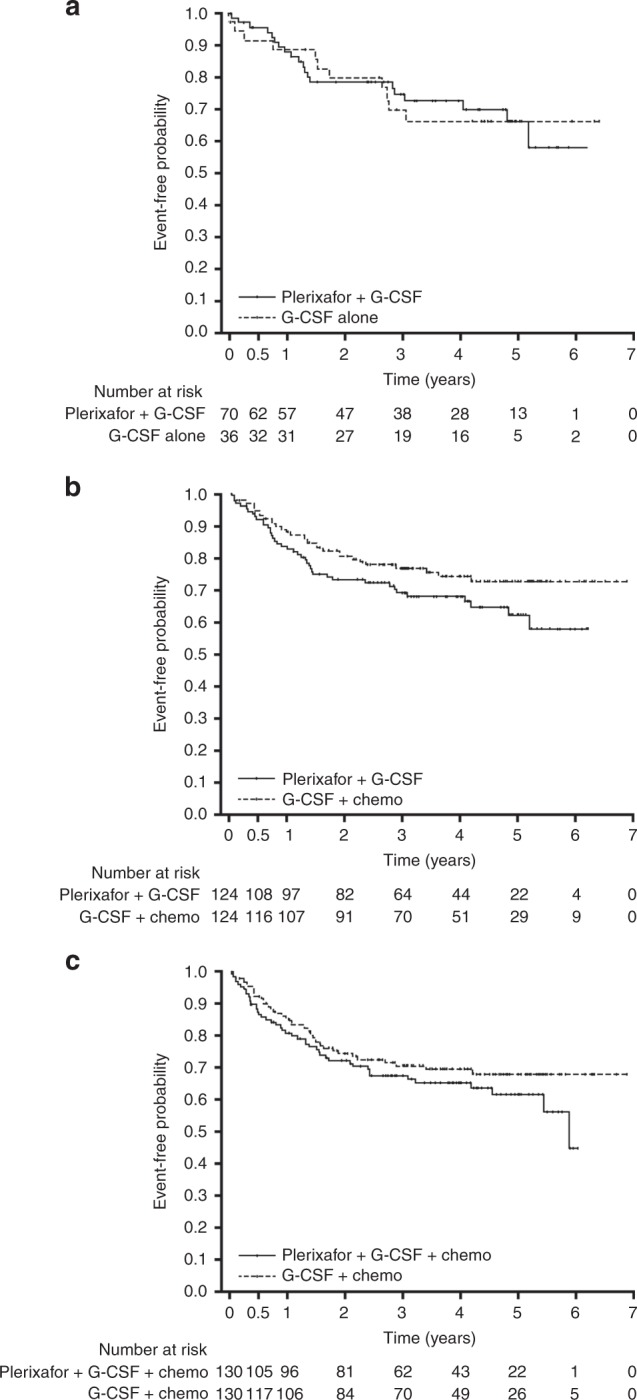
Overall survival for each of the comparison groups, **a** G-CSF + plerixafor versus G-CSF alone (comparison 1); **b** G-CSF + plerixafor versus G-CSF + chemotherapy (comparison 2); and **c** G-CSF + plerixafor + chemotherapy versus G-CSF + chemotherapy (comparison 3)

Due to censoring median follow-up and interquartile ranges were not calculable for some comparison groups. Kaplan–Meier estimates of OS for the G-CSF + plerixafor versus the G-CSF alone group (comparison 1, Fig. 3a) were similar. Similarly, Kaplan–Meier estimates of OS for the G-CSF + plerixafor group versus G-CSF + chemotherapy group (comparison 2, Fig. 3b) were also similar, although the G-CSF + plerixafor group trended lower after 0.5 years compared with the G-CSF + chemotherapy group. For the G-CSF + plerixafor + chemotherapy group compared with the G-CSF + chemotherapy group OS was also similar between groups (comparison 3, Fig. 3c). As the upper limit of the HR was >1.3, based on predetermined boundaries, non-inferiority of plerixafor was not demonstrated for any of the comparison groups. Estimated OS at 3 years are shown in Table 3.

#### Cumulative incidence of relapse rates

**Fig. 4:**
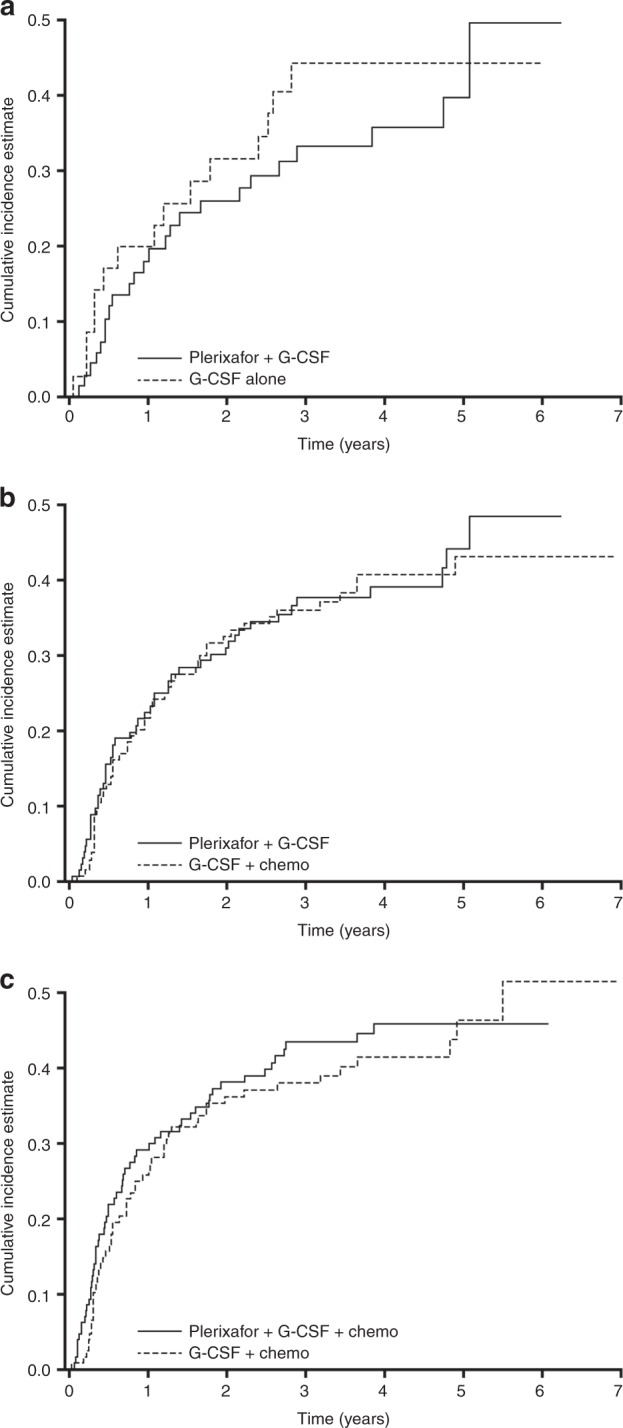
Cumulative incidence of relapse for each of the comparison groups, **a** G-CSF + plerixafor versus G-CSF alone (comparison 1); **b** G-CSF + plerixafor versus G-CSF + chemotherapy (comparison 2); and **c** G-CSF + plerixafor + chemotherapy versus G-CSF + chemotherapy (comparison 3)

Cumulative incidence of relapse rates at 3 years are shown in Table 3. The CIR trended higher in the G-CSF alone group compared with the G-CSF + plerixafor group until year 5. However, the CIR was similar between the G-CSF + plerixafor versus the G-CSF + chemotherapy group, and was similar between the G-CSF + plerixafor + chemotherapy group versus the G-CSF + chemotherapy group (Fig. 4).

### Secondary outcomes

#### Post transplantation

Adverse events occurring in more than one patient in any treatment group up to 100 days post first transplantation are shown in Table 4. Infections and infestations were the most common standard organ class complication in all plerixafor and comparator groups.

**Table 4:** Adverse events occurring in more than one patient in any treatment group up to 100 days post first transplantation

	Comparison 1		Comparison 2		Comparison 3	
Adverse event standard organ class, *n* (%) preferred term	G-CSF + plerixafor (*N* = 70)	G-CSF alone (*N* = 36)	G-CSF + plerixafor (*N* = 124)	G-CSF + chemotherapy (*N* = 124)	G-CSF + plerixafor + chemotherapy (*N* = 130)	G-CSF + chemotherapy (*N* = 130)
Any adverse event up to 100 days post transplantation	37 (52.9)	19 (52.8)	66 (53.2)	62 (50.0)	55 (42.3)	68 (52.3)
Infections and infestations	32 (45.7)	13 (36.1)	56 (45.2)	51 (41.1)	42 (32.3)	59 (45.4)
Bacterial infections	16 (22.9)	8 (22.2)	29 (23.4)	31 (25.0)	33 (25.4)	36 (27.7)
Blood and lymphatic disorders	5 (7.1)	3 (8.3)	8 (6.5)	5 (4.0)	2 (1.5)	3 (2.3)
Gastrointestinal disorders	2 (2.9)	1 (2.8)	4 (3.2)	3 (2.4)	3 (2.3)	4 (3.1)
General disorders and administration site disorders	8 (11.4)	1 (2.8)	11 (8.9)	14 (11.3)	21 (16.2)	17 (13.1)
Metabolism and nutritional disorders	0	0	0	2 (1.6)	0	2 (1.5)
Renal and urinary disorders	1 (1.4)	1 (2.8)	2 (1.6)	1 (0.8)	3 (2.3)	2 (1.5)
Vascular disorders	0	1 (2.8)	0	1 (0.8)	2 (1.5)	2 (1.5)
Cardiac disorders	0	0	0	2 (1.6)	1 (0.8)	3 (2.3)
Nervous system disorders	0	0	1 (0.8)	0	2 (1.5)	0
Respiratory, thoracic, and mediastinal disorders	0	0	0	1 (0.8)	3 (2.3)	1 (0.8)
Skin and subcutaneous disorders	0	0	0	0	2 (1.5)	0

*G-CSF* granulocyte-colony stimulating factor

Engraftment was reported in ≥88% of patients in each treatment group of the three comparisons. Similarly, engraftment of both platelets and neutrophils was reported in ≥82% of patients in each treatment group. The median number of days to reach a platelet count of ≥20 × 10^9^/L was 14 days for each of the plerixafor groups and 12–13 days for the respective comparator groups. The median number of days to reach a neutrophil count of ≥0.5 × 10^9^/L was 11–12 days for each group.

## Discussion

This study was a postapproval measure to investigate clinical disease outcome as a surrogate of potential enhancement of tumor cell release when combining G-CSF + plerixafor. The study was not designed to evaluate the benefit of stem cell mobilization with plerixafor compared with other methods. In general, no major differences were observed in PFS, OS, or CIR between the plerixafor and the comparison groups.

In line with the plerixafor label all patients in the study had to be poor mobilizers, either as proven poor mobilizers, based on their mobilization history, or as predicted poor mobilizers through exposure to high-dose chemotherapy. However, despite propensity score matching, which tried to balance the groups, there were still substantially more proven poor mobilizers in the plerixafor cohorts, which may have led to an imbalance between comparison groups. Consistently the median pre-apheresis CD34+ cell counts in the plerixafor cohorts during the current mobilization at the predicted time of peak mobilization were lower compared with comparison cohorts. Therefore, it appears that the groups were not well balanced for disease and HSCT risk.

Others have shown that lymphoma patients’ poor mobilization of autologous stem cells, defined as the inability to obtain ≥1 × 10^6^ CD34+ cells/kg body weight, resulted in substantially lower PFS and OS compared with good mobilizers [14, 15]. Therefore, in our study, the higher proportion of proven poor mobilizers in the plerixafor groups may, at least in part, explain the PFS, OS, and CIR results versus those in the comparison groups. Furthermore, there are two confounders, which might have skewed the analysis of the data in the current study. Firstly, poor mobilization could be an indicator of more severe or prognostically worse disease. Therefore, the post-plerixafor outcomes trending toward slightly worse OS, PFS, and CIR could be the result of more advanced lymphoma disease and not a trend toward inferiority of plerixafor-mobilized grafts. Secondly, data on the lymphoma biology were not available, which does not allow the confirmation that the plerixafor and comparator groups were balanced in relation to the stage of the lymphoma disease.

Engraftment was reported in ≥88% of patients in each of the three comparator cohorts and the median number of days to achieve the target levels of platelets and neutrophils was similar for all cohorts (12–13 days and 11–12 days, respectively), which was in line with findings from a previous study [16].

In support of our findings, results from a 5-year, long-term, phase 3, follow-up study (not restricted to poor mobilizers) suggested that the use of G-CSF + plerixafor did not have a negative outcome on PFS and OS in patients with lymphoma, with more than a half of patients with lymphoma remaining alive 5 years post transplantation [17].

Propensity scores are increasingly used in nonrandomized studies to assess marginal treatment effects and to reduce potential bias and imbalances of baseline covariates [12]. The assumption of propensity scores is that none of the unmeasured covariates affecting treatment choice confound the association between treatment and outcome [18]. Unobserved differences between groups cannot be adjusted by propensity scores; therefore, this is both a source of potential bias and a limitation of propensity scores [19]. Despite the modest sample size in the comparisons resulting from propensity scoring matching, in our study, an adequate balance for certain key disease characteristics collected in the database was maintained. However, we acknowledge that some key factors affecting mobilization outcome were unavailable in the database, such as, the number of rounds and the type of chemotherapy administered, and for those who received radiotherapy the extension of irradiated fields. These limitations in data collection may have had a major impact on the outcomes in our study.

Moreover, in line with the reimbursement criteria for the drug and the obligation on some clinicians to closely manage treatment costs [20], plerixafor may have been selectively given to patients who were the poorest mobilizers at highest risk, and this may have been a factor in the trend for slightly worse outcomes in the plerixafor-treated groups. Conversely, only patients with successful mobilization were included in the study, this may have inadvertently introduced selection bias in favor of the non-plerixafor cohorts. Furthermore, our study was not adequately powered, as there were small to moderate numbers of patients in each cohort after propensity score matching. Although every effort was made to remove potential biases in this study, biases may not have been removed completely due to the observational nature of the data.

## Conclusions

Although non-inferiority of the plerixafor groups could not be formally shown, PFS, OS, and CIR were numerically similar between comparators. Results from this study should be interpreted with caution due to a number of limitations. These include an imbalance between treatment groups due to the greater proportion of proven poor mobilizers in the groups treated with plerixafor, the limited scope of the EBMT database, the lack of power, and the observational nature of the study. In particular, a higher proportion of patients treated with plerixafor failed to mobilize sufficient CD34+ cells at the predicted time. Therefore, these patients represent a group that is likely predisposed to worse outcomes. Infections and infestations were the most common standard organ class posttransplant complication for plerixafor and comparator cohorts. Without plerixafor treatment, it is likely that some patients may not have proceeded to transplantation. Altogether, this study does not provide evidence that plerixafor mobilization is associated with an increased risk of relapse or post-transplant toxicities in patients undergoing HSCT for lymphoma.

## Data Availability

Qualified researchers may request access to patient level data and related study documents including the clinical study report, study protocol with any amendments, blank case report form, statistical analysis plan, and dataset specifications. Patient level data will be anonymized and study documents will be redacted to protect the privacy of trial participants. Further details on Sanofi’s data sharing criteria, eligible studies, and process for requesting access can be found at: https://www.clinicalstudydatarequest.com.
